# Supplementary material for: Lentiviral vectors escape innate sensing but trigger p53 in human hematopoietic stem and progenitor cells
Source: EMBO Mol Med. 2017 Jun 30;9(9):1198–211. doi: 10.15252/emmm.201707922 (PMC5582409; doi:10.15252/emmm.201707922)
Supplement: Supplementary file 1 — Appendix [file EMMM-9-1198-s001.pdf]

Appendix Table of Contents:

Appendix Table S1

Appendix Table S2

Appendix Table S3

Appendix Table S4

Appendix Table S5

Supplementary Methods

Supplementary References

Appendix Table S1. Number of genes changing and differentially expressed over time under *p*-value ≤0.05.

Condition	Genes changing over time
Untreated	5883
Poly(I:C)	9496
Bald LV	7307
Lab LV	7080
Purified LV	7248
Inactivated purified LV	7512
**Condition**	**Genes differentially expressed between conditions**
Untreated Vs. Poly (I:C)	2691
Untreated Vs. Lab LV	645
Untreated Vs. Purified LV	397
Bald LV Vs. Lab LV	321
Inactivated Purified LV vs. Purified LV	281

Appendix Table S2. Significance, in the context of the comparison between Lab LV and Bald LV, of the top 40 genes activated by poly(I:C) in HSPC.

Gene	*p* value
IFI6	0.662955
TVP23A	0.407102
ISG15	0.964351
IFI44L	0.525873
IFI44	0.812837
IFI35	0.955392
ISG20	0.020014
DDX60	0.840026
HERC6	0.973159
LGALS3BP	0.753786

HLA-F	0.935258
MX1	0.904987
PARP12	0.924286
EPSTI1	0.971483
SAT1	0.216319
OAS3	0.980706
IFITM1	0.861568
HCP5	0.831475
IRF7	0.80476
XAF1	0.796685
DHX58	0.764003
H19	0.217731
ODF3B	0.569558
IFI27	0.456448
TRIM22	0.00061
CSAG3	0.369604
USP18	0.95428
KLHDC7B	0.960367
MX2	0.948736
OAS1	0.983136
HLA-DQA1	0.566047
CCL5	0.503809
GDF11	0.74255
ARG2	0.864504
OPTN	0.953236
OAS2	0.971224
GP1BA	0.714797
USP41	0.784604
LAMC2	0.256181
STAT1	0.586429
PLSCR1	0.496799

IFITM3	0.441251
HELZ2	0.695985
ACO1	0.579893
DDX58	0.86283
C19orf66	0.724447
PARP9	0.950643
OASL	0.832593
CXCL11	0.182701

Appendix Table S3. Cell counts of in vivo experiments.

	N Cell Counted at TD	N Cell Counted at TP	N Cell Infused	Source
Empty LV	890000	816666	80000	CB
LV	1240000	1390000	80000	
Empty LV	840000	690000	80000	
LV	1160000	900000	80000	
Bald DMSO	700000	904000	80000	
LV DMSO	700000	840000	80000	
Bald KU	800000	957000	80000	
LV KU	800000	1030000	80000	
Bald DMSO	625000	1000000	80000	
LV DMSO	625000	600000	80000	
Bald KU	625000	800000	80000	
LV KU	625000	600000	80000	
Inact LV	1750000	3710000	514285	mPB
Clinical-grade LV	1750000	3050000	422857	
Bald	800000	1744000		CB
LV	800000	1216000		
Bald DMSO	2000000	2200000	500000	CB
LV DMSO	2000000	2075000	500000	

Appendix Table S4. List of anti-human antibodies used in this study.

**Antibody for FACS**	**Fluorochrome**	**Dilution**	**Clone**	**Company**	**Code**
hCD235a	APC	1:25	GA-R2	BD Biosciences	551336
hCD33	BV421	1:25	WM53	BD Biosciences	562854
Anti human FCR Blocking		1:50		Miltenyi Biotec	120-000-442
Anti murine FCR Blocking		1:100	2.4G2	BD Pharmigen	553142
hCD45	APC-Cy7	1:33	HI30	eBiosciences	47-0459-42
hCD19	PE	1:25	SJ25C1	BD Biosciences	345789
hCD33	PeCy7	1:25	P67.6	BD Biosciences	333952
hCD3	APC	1:25	UCHT1	BD Biosciences	555335
hCD13	BV	1:25	WM15	BD Biosciences	562596
hCD34	PeCy7	1:25	8G12	BD Biosciences	348811
hCD38	V450	1:25	HB7	BD Biosciences	646851
hCD90	APC	1:25	5E10	BD Biosciences	559869
hCD45RA	VioBlue	1:10	T6D11	Miltenyi Biotec	130-095-464
hCD133	PE	1:15	293C3	Miltenyi Biotec	130-090-853
hCD38	APC	1:10	IB6	Miltenyi Biotec	130-092-261
Ki-67	PE		B56	BD Bioesciences	556027

hCD90	Brillant Violet	1:30		Biolegend	328122
Annexin V	Pacific Blue	1:20		Biolegend	640918
Hoechst				Thermo Fisher	H3570
7-AAD				Sigma-Aldricht	A9400
**Antibody for WB**	**Produced in**	**Dilution**	**Clone**	**Company**	**Code**
p53Ser15	Rabbit	1:1000	Polyclonal	Cell signaling Technology	284S
p53	Mouse	1:200	DO-1	Santa Cruz	Sc126
p21	Rabbit	1:1000	12D1	Cell signaling Technology	2947
γ-H2AX	Rabbit	1:1000	20E3	Cell signaling Technology	9718
pSer1981ATM	Rabbit	1:1000	D25E5	Cell signaling Technology	13050
ATM	Rabbit	1:1000	D2E2	Cell signaling Technology	2873

Appendix Table S5. List of primers used in this study.

Primers for GE	Method	Company	Code
hCDKN1A (p21)	RT-PCR	Thermo Fisher	Hs00355782_m1
hHPRT1	RT-PCR	Thermo Fisher	Hs01003267_m1
hIRF7	RT-PCR	Thermo Fisher	Hs01014809_g1
hPHLDA3	RT-PCR	Thermo Fisher	Hs00385313_m1
hISG15	RT-PCR	Thermo Fisher	Hs01921425_s1

hOAS1	RT-PCR	Thermo Fisher	Hs00973637_m1
hDDB2 (p48)	RT-PCR	Thermo Fisher	Hs03044953_m1
hBBC3 (PUMA)	RT-PCR	Thermo Fisher	Hs00248075_m1
mIrf7	RT-PCR	Thermo Fisher	Mm00516788_m1
mOas1	RT-PCR	Thermo Fisher	Mm00836412_m1
mIfit1	RT-PCR	Thermo Fisher	Mm00515153_m1
mIsg15	RT-PCR	Thermo Fisher	Mm01705338_s1
mHprt	RT-PCR	Thermo Fisher	Mm01545399_m1
mCdkn1a	RT-PCR	Thermo Fisher	Mm04207341_m1
**Primer for VCN**	**Sequence (5' → 3')**	**Method**	**Reference**
LV-Sense	TACTGACGCTCTCGCACC	RT-PCR/ddPCR	(Matrai et al, 2011)
LV-Antisense	TCTCGACGCAGGACTCG		
LV-Probe	ATCTCTCTCCTTCCTTCTAGCCTC		
RT-LV (ΔU3 sense)	TCACTCCCAACGAAGACAAGATC		
RT-LV (Gag antisense)	GAGTCCTGCGTCGAGAGAG		
RT-RV (ΔU3 sense)	CGAGCTCAATAAAAGAGCCCAC	ddPCR	
RT-RV (PBS antisense)	ACAGATAGGTTGCTGGCCAG	ddPCR	
hTert fw	GGCACACGTGGCTTTTCG	RT-PCR/ddPCR	(Lombardo et al, 2007)
hTert rev	GGTGAACCTCGTAAGTTTATGC		

	AA		
hTert Probe	TCAGGACGTCGAGTGGACACG GTG		
2LTR Fw (2Junct)	CAGTGTGGAAAATCTCTAGCAG TAC	ddPCR	(Petrillo et al, 2015)
2LTR Rev (J2 Rev)	GCCGTGCGCGCTTCAGCAAGC		

Supplementary Methods

Cells and transductions

K562 and Hela cell lines were plated in complete Iscove's modified Dulbecco's medium (Euroclone). HL-60 cells were grown and transduced in complete RPMI. HCT-116 cells were grown and transduced in complete Dulbecco's Modified Eagle Medium (DMEM). Human CD34$^+$ hematopoietic stem and progenitor cells (HSPC) and CD4$^+$ T cells were isolated through magnetic bead selection according to manufacturer's instructions (Milteny) from umbilical cord blood (CB) collected upon informed consent from healthy volunteers according to the Institutional Ethical Committee approved protocol (TIGET01). Otherwise, CB and bone marrow (BM)-derived CD34$^+$ were directly purchased from Lonza. G-CSF mobilized peripheral blood (mPB)-CD34$^+$ cells were purchased from hemacare. Murine Lin$^-$ cells were isolated through magnetic bead selection according to the manufacturer's instructions (Miltenyi) from bone marrow of euthanized C57/BL6 mice.

Soon after purification or thawing, human CB CD34$^+$ cells were plated in culture at a concentration of 1×10^6 cells/ml in CellGro Serum-Free Medium (CellGenix) or StemSpan (STEMCELL TECHNOLOGIES) supplemented with penicillin (100 IU/ml), streptomycin (100 µg/ml) and 2% glutamine in the presence of human cytokines (Interleukin-6 (IL-6) (20 ng/ml), thrombopoietin (TPO) (20 ng/ml), Stem Cell Factor

(SCF) (100 ng/ml), and Flt3 ligand (Flt3-L) (100 ng/ml) (PeproTech) for O/N prestimulation. BM and mPB-CD34+ were plated in retronectine-coated plates in StemSpan or CellGro respectively supplemented with penicillin (100 IU/ml), streptomycin (100 µg/ml) and 2% glutamine in the presence of human cytokines Interleukin3 (IL-3) (100 ng/ml), thrombopoietin (TPO) (100 ng/ml), Stem Cell Factor (SCF) (300 ng/ml), and Flt3 ligand (Flt3-L) (300 ng/ml) (PeproTech) for O/N prestimulation. After 18-24 hours of stimulation $CD34^+$ cells were transduced at the indicated MOI. For the clinical standard double-hit transduction protocol, cells were washed 16 hours after the first vector exposure, left to recover in cytokine-supplemented medium for 10 hours and re-exposed to the second hit of vector for another 16 hours before transplantation. Transduction with the AAV6 vectors were performed at an MOI of 10000 as calculated by titration of the vector preparation expressed in vector genome per mL (vg/mL). All the transductions were performed at 1×10^6 cells/ml concentration. In the experiment with antiretroviral inhibitors, the drugs were added together with the vector. Raltegravir and 3TC were used at 10 µM, and AZT at 25 µM. In the ATM inhibition experiment single hit of the ATM inhibitor KU55933 (Selleck Chemicals) was added on the cells 2 hours before the transduction at 10 µM concentration. All cells were maintained in a 5% CO2 humidified atmosphere at 37°C.

RNA-Seq data generation and analysis

Total RNA was extracted with the RNeasy Plus Micro kit (Qiagen) according manufacturer's indications. RNA integrity was analyzed with the Agilent 2100 Bioanalyzer (Agilent Technologies, Santa Clara, CA). Libraries, prepared starting from 100ng of total RNA/sample with the Illumina TruSeq RNA Sample Prep kit v2 procedure, were quantified by the Qubit BR assay (Life Technologies, Illkirch,

France) and the Agilent 2100 Bioanalyzer. Sequencing was performed on the Illumina HiSeq 2000 platform using SBS 2x100PE protocol. In average for each sample we obtained 30M reads .Each gene was then characterized by the total number of reads overlapping it. Normalization and differential gene expression was evaluated using linear models implemented in R/Bioconductor limma package(Smyth, 2004). Pathway analysis was initially performed using EnrichrR platform (Chen et al, 2013), most of the advanced network modeling was performed using Cytoscape (v3.2.0). The complete RNA-Seq dataset is available at NCBI, accession number GSE92652.

FACS

All cytometric analyses were performed using the FACS Canto III and LSRFortessa instruments (BD Biosciences, San Jose, CA) and analyzed with the FACS Express software (De Novo Software, Glendale, CA). GFP expression in transduced cells was measured 5-7 days post-transduction. To exclude dead cells from the analysis, cells were washed and resuspended in PBS containing 10 ng/ml 7-aminoactinomycin D (7-AAD, Sigma-Aldrich). The apoptosis assays were performed with the Annexin V Apoptosis Detection Kit I (BD Pharmigen) according to the manufacturer's instructions and 48 hours after transduction, if not otherwise indicated. The cell proliferation assay was performed with the Cell Proliferation Dye eFluor 670 (eBioscience) according to the manufacturer's instructions. To calibrate the cytometer, rainbow beads (Spherotech) were used to set the dye signal in the different days of the analysis. The cell cycle analysis was performed by Ki67 (BD Pharmigen) and Hoechst (Invitrogen) staining as previously described (Lechman et al, 2012) 48 hours after the transduction. (for all antibodies see **Appendix Table S4**).

RNA, DNA and Proteins

Total RNA was extracted with the RNeasy Plus Micro kit or RNeasy Micro Kit (Qiagen) and Reverse transcription was performed using SuperScriptVILO cDNA Synthesis Kit (ThermoFisher Scientific) according to manufacturers' instructions. Gene expression analysis was performed by Taqman probe (Thermo Fisher, **Appendix Table S5**) as previously described (Petrillo et al., 2015), human HPRT1 or murine Hprt were used to normalize the total quantity of human or mouse cDNA input respectively. See Table S6 for the complete list of Taqman Probe reagents.

Vector Copy Number (VCN)

For VCN, total DNA was extracted using a Maxwell 16 instrument (Promega) or Blood & Cell Culture DNA micro kit (Qiagen). Copy Number of the integrated lentiviral vector were assessed as previously described (Lombardo et al., 2007; Petrillo et al., 2015; Santoni de Sio et al., 2008) or by digital droplet PCR (dd-PCR), (BIO-RAD, California, USA) according to the manufacturers' instructions using hTERT gene as normalizer. Copy Number of the total lentiviral DNA (integrated and non-integrated) were performed as previously described (Matrai et al., 2011) at three days post-transduction. Copy Number of the reverse transcribed retroviral vector genome (both integrated and non-integrated) was performed by dd-PCR discriminating it from plasmid carried over from the transient transfection using the following primers: RT-RV; ΔU3 sense: 5'-CGAGCTCAATAAAAGAGCCCAC-3', PBS antisense: 5'-GAGTCCTGCGTCGGAGAGAG-3'. The amount of human DNA loaded in the reaction was quantified with a qPCR or ddPCR designed to amplify the hTERT gene as described (Lombardo et al., 2007). The 2LTR circles copy number was performed in dd-PCR with previously described primers (Petrillo et al., 2015). See

Appendix Table S5 for the complete list of primers. Unless otherwise specified, copy numbers are expressed as amplicon copies per cell (diploid genome).

Western Blot

Western Blot was performed as previously described (Kajaste-Rudnitski et al., 2006; Petrillo et al., 2015). Samples were subjected to SDS-PAGE on Bolt 4-12% Bis-Tris Plus gels (ThermoFisher, CAT # NW04120BOX) and transferred to PVDF membrane by electroblotting. See **Appendix Table S4** for the complete list of the antibodies.

Supplementary References

Chen EY, Tan CM, Kou Y, Duan Q, Wang Z, Meirelles GV, Clark NR, Ma'ayan A (2013) Enrichr: interactive and collaborative HTML5 gene list enrichment analysis tool. *BMC Bioinformatics* **14:** 128

Lechman ER, Gentner B, van Galen P, Giustacchini A, Saini M, Boccalatte FE, Hiramatsu H, Restuccia U, Bachi A, Voisin V, Bader GD, Dick JE, Naldini L (2012) Attenuation of miR-126 activity expands HSC in vivo without exhaustion. *Cell Stem Cell* **11:** 799-811

Lombardo A, Genovese P, Beausejour CM, Colleoni S, Lee YL, Kim KA, Ando D, Urnov FD, Galli C, Gregory PD, Holmes MC, Naldini L (2007) Gene editing in human stem cells using zinc finger nucleases and integrase-defective lentiviral vector delivery. *Nat Biotechnol* **25:** 1298-1306

Matrai J, Cantore A, Bartholomae CC, Annoni A, Wang W, Acosta-Sanchez A, Samara-Kuko E, De Waele L, Ma L, Genovese P, Damo M, Arens A, Goudy K, Nichols TC, von Kalle C, MK LC, Roncarolo MG, Schmidt M, Vandendriessche T, Naldini L (2011) Hepatocyte-targeted expression by integrase-defective lentiviral vectors induces antigen-specific tolerance in mice with low genotoxic risk. *Hepatology* **53:** 1696-1707

Petrillo C, Cesana D, Piras F, Bartolaccini S, Naldini L, Montini E, Kajaste-Rudnitski A (2015) Cyclosporin a and rapamycin relieve distinct lentiviral restriction blocks in hematopoietic stem and progenitor cells. *Mol Ther* **23:** 352-362

Smyth GK (2004) Linear models and empirical bayes methods for assessing differential expression in microarray experiments. *Stat Appl Genet Mol Biol* **3:** Article3
