# Supplementary material for: Lentiviral vectors escape innate sensing but trigger p53 in human hematopoietic stem and progenitor cells
Source: EMBO Mol Med. 2017 Jun 30;9(9):1198–211. doi: 10.15252/emmm.201707922 (PMC5582409; doi:10.15252/emmm.201707922)
Supplement: Supplementary file 2 — Expanded View Figures PDF [file EMMM-9-1198-s002.pdf]

Expanded View Figures

Figure EV1. Characterization of the transcriptional responses in HSPC.

A Principal component analysis (PCA) plot. The samples from the RNA-seq experiment are shown in the 3D plane spanned by their first three principal components. The first three principal components accounted for more than the 69% of explained variance among the data set.

B Bar chart of the pathway enrichment analysis performed with WikiPathways database on the significant ($P \leq 0.05$) differentially expressed genes over time in the untreated condition or between the Poly(I:C) and Mock conditions. The length of the bar represents the significance (*P*-value Ranking) of that specific pathway.

C p21 mRNA levels were measured after 48 h from the transduction of CB-CD34^{+} with an MOI of 100 PGK-GFP SIN LV (LV), MOI of 100 clinical-grade LV (clinical LV), or to p24 equivalent of Env-less (Bald) or inactivated clinical LV as controls (inactivated clinical LV) (mean ± SEM, n = 3).

D p21 mRNA levels 48 h post-transduction and vector copy numbers (VCN) 14 days after transduction were measured in CB-CD34^{+} exposed to increasing MOI of PGK-GFP SIN LV (LV).

E p21 mRNA levels in CB-CD34^{+} cells 5 days after transduction with an MOI 100 of LV, IDLV, or p24 equivalent of Bald or Empty LV as controls. Results are shown respect to Bald for each cell type set to value 1 (Red threshold) (mean ± SEM, n = 6).

F p21 mRNA levels 48 days post-transduction in different human and murine cell lines or primary cells exposed to PGK-GFP SIN LV or p24 equivalent of Bald as control. p21 mRNA was measured 48 h after the transduction and normalized to HPRT1 for human cells or Hprt for Murine cells. Results are shown respect to Bald for each cell type set to value 1 (Red threshold) (mean ± SEM, n = 17 for hu-CD34^{+}, n = 8 for mu-HSPC, n = 3 for CD4^{+} T cells and HCT116, n = 4 for HL60, n = 2 for K562 and Hela).

G VCN per cell were performed 14 days after exposure of sorted CB-CD34^{+} to PGK-GFP SIN LV (LV), at MOI 100 or p24 equivalents of Env-less (Bald) as indicated (mean ± SEM, n = 5 for 133^{+}38^{-}, n = 8 for 133^{+}38^{int} and 133^{+}38^{hi}).

H Copy number per cell of total viral DNA 3 days after the transduction of human CB-CD34^{+} exposed to an MOI of 100 PGK-GFP SIN LV (LV), integrase-defective LV (IDLV), p24 equivalent genome-less (Empty LV), or Env-less (Bald) (mean ± SEM, n = 4).

I, J Vector copy number results and WB for p53 and p21 of CB-CD34^{+} transduced with an MOI of 100 of PGK-GFP SIN LV (LV), or Env-less (Bald) as control. Transductions were performed in the presence of the integrase inhibitor Raltegravir (Ral), or the reverse-transcriptase inhibitor Lamivudine (3TC). Control cells were transduced in DMSO or kept untreated (Mock). VCN and WB were performed 14 days and 48 h after transduction, respectively (mean ± SEM, n = 4, I; n = 1, J).

K Representative FACS plot of CB-CD34^{+} cells 48 h after the transduction with PGK-BFP LV, IDLV, or Bald as control and stained with anti-p21 antibody.

L Immunofluorescence of CB-CD34^{+} cells stained for γH2AX (left panel), 12 hours after the transduction with the indicated vector or camptothecin (CPT) exposure, and for p21 (right panel) 48 h after the transduction with PGK-mCHERRY LV, IDLV, a p21 overexpressing LV (p21 OE) or Bald as a control (representative image of n = 2).

M Percentage of GFP positive CB-CD34^{+} transduced with PGK-GFP SIN LV at MOI 100 (LV), or exposed to p24 equivalent of Env-less (Bald) vector as control, cells were also transduced with an MOI 100 of a PGK-GFP SIN RV or to an MOI 10,000 of PGK-GFP Adeno-associated Vector (AAV6). GFP expression was assessed at FACS 5 days after transduction for the integrating vector, and 2 days post-TD for the AAV6 (mean ± SEM, n = 9).

N Vector copy number results of CB-CD34^{+} transduced with PGK-GFP SIN LV or PGK-GFP SIN RV (RV) at MOI 100 or exposed to p24 equivalent of Env-less (Bald) vector as control. Copy per cell of total viral DNA was assessed 3 days after transduction, while VCN on the integrated vectors were performed 14 days after transduction (mean ± SEM, n = 9).

O, P (O) Comparison of the nuclear import efficiency and (P) total viral DNA 3 days after transduction with LV or ΔcPPT. Nuclear import efficiency is reported as the percentage of 2LTR circles on the total reverse-transcribed viral DNA. The same analysis was performed in 293T cells (upper panel) and in human CBCD34^{+} cells (MOI 100) (lower panel) (mean ± SEM, n = 4 for 293T, n = 12 for CD34^{+}, Mann–Whitney test, *P = 0.0286, ***$P < 0.0001$, O), (mean ± SEM, n = 4 for 293T, n = 12 for CD34^{+}, Mann–Whitney test, P).

Q Interferon-stimulated gene (ISG) expression in human CB-CD34^{+} transduced with an MOI of 100 of PGK-GFP SIN RV (γ-RV), or heat-inactivated RV (Inact RV) as control. Transductions were performed in the presence of the integrase inhibitor Raltegravir (Ral), reverse-transcriptase inhibitor azidothymidine (AZT), or a neutralizing antibody against the human IFNα receptor (α-IFNAR). FACS analysis of GFP expression was performed 2 days after the transduction (upper panel). ISGs (IRF7, OAS1, ISG15) were measured 48 h post-transduction. Expression levels were normalized to HPRT1 and shown respect to Bald set to value of 1 (lower panel) (mean ± SEM, n = 6 for Inact RV, RV, RV + Ral, RV + AZT, n = 2 for RV + αIFNAR).

R ISG15 and p21 mRNA levels in CB-CD34^{+} cells exposed to 1,000 U/ml of human IFNα for 24 h and transduced for 48 h either with an MOI 100 of a PGK-GFP LV or p24 equivalent of Bald as a control (mean ± SEM, n = 2).

S ISG (IFIT1, IRF7, OAS1, and ISG15) induction in murine HSPC transduced with an MOI of 100 of PGK-GFP SIN LV (LV), PGKGFP SIN RV (RV), or a p24 equivalent of Env-less (Bald) 48 h post-transduction. Cells were also left untreated as control in the mock condition. Expression levels were normalized to HPRT1 and shown respect to controls set to value of 1 (mean ± SEM, n = 3 for Mock, n = 8 for bald and LV, n = 4 for RV).

Source data are available online for this figure.

▶

© 2017 The Authors

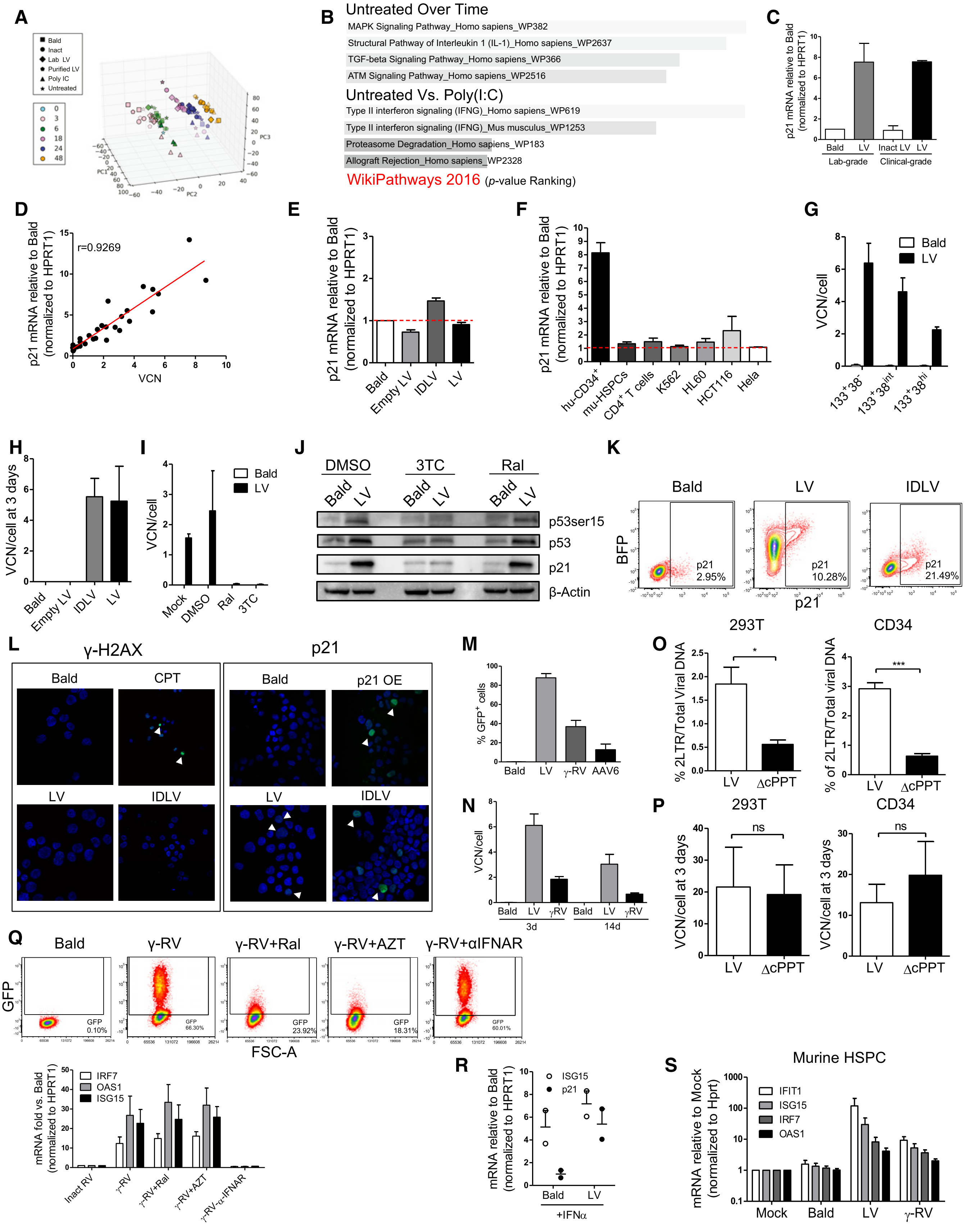

Figure EV1.

© 2017 The Authors

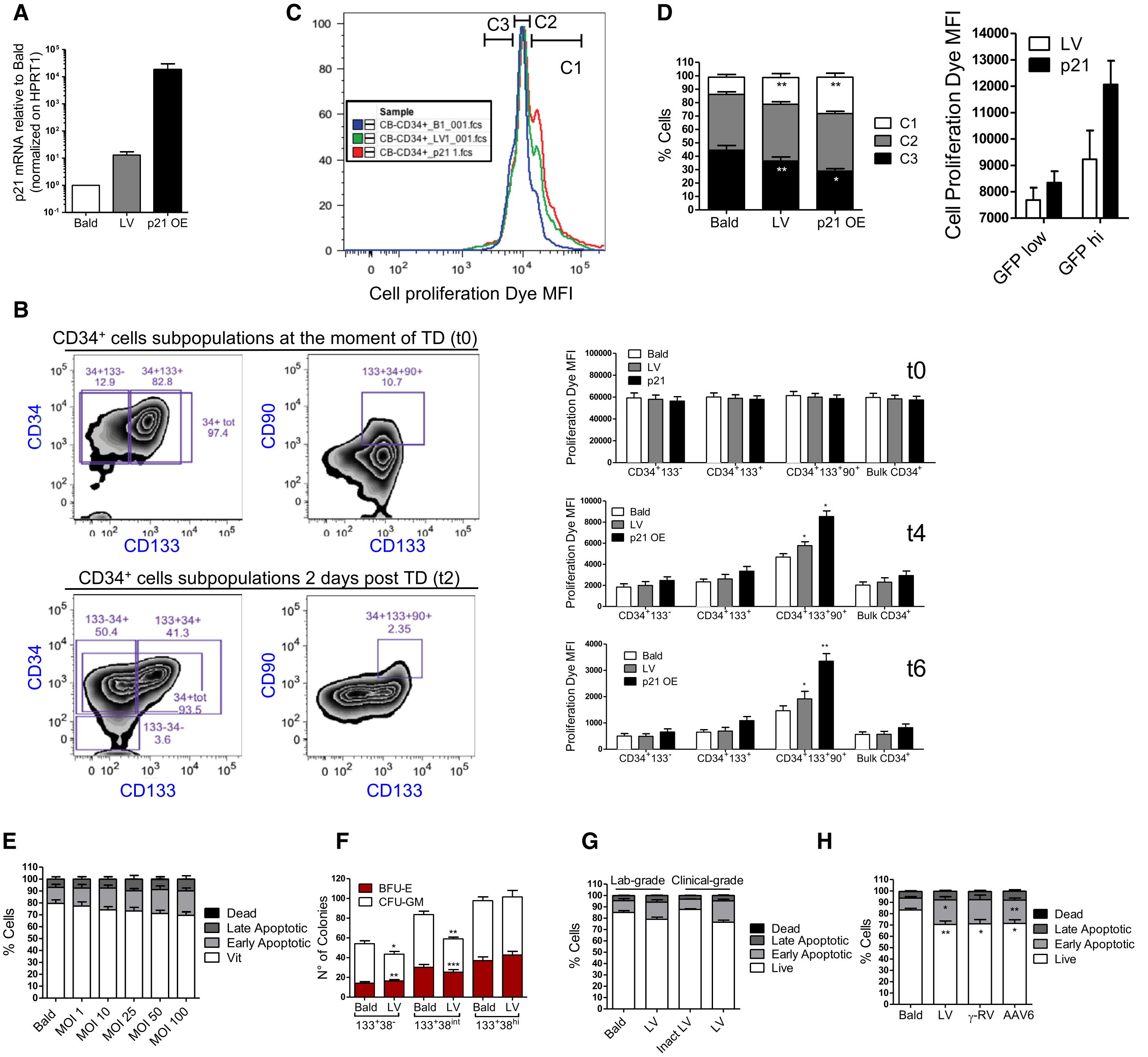

Figure EV2. *In vitro* impact of LV.

A p21 expression levels 48 h after the transduction of human CB-CD34$^+$ exposed to an MOI of 100 PGK-GFP SIN LV (LV), p21 overexpressing LV (p21 OE) or p24 equivalent of Bald (mean ± SEM, n = 8).

B Representative gating strategy to analyze the CD34$^+$ cells treated as in Fig 2A. The upper panel shows the percentage of CD34$^+$, CD133$^+$, and CD90$^+$ cells at the moment of transduction, the lower panel reports the same markers 2 days after the transduction. In the right panels, the bar graphs show the MFI of the cell proliferation dye at the moment of transduction or 4 and 6 days after the transduction within each subpopulation (mean ± SEM, n = 8, Wilcoxon matched pairs test, *P = 0.0313 for t4, *P = 0.0156 and **P = 0.0078 for t6).

C, D (C) Representative histograms and (D) bar graphs of cells treated as in Fig 2A at 2 days after transduction. The analyses were performed to assess the percentage of CB-CD34$^+$ cells that are contained within each gate (C1, C2, C3) for LV, p21, and Bald conditions. The cells within the C1 gate have proliferated less, while the one in the C3 have proliferated more (mean ± SEM, n = 8, Wilcoxon signed-rank test, *P = 0.0142, **P = 0.0078, left panel) (mean ± SEM, n = 6, right panel).

E Annexin V staining for apoptotic cells was performed 2 days after transduction of human CB-CD34$^+$ exposed to increasing MOI of PGK-GFP SIN LV (LV) (mean ± SEM, n = 6).

F After the sorting and transduction as indicated in Fig 1F, CB-CD34$^+$ cells were plated in semisolid, cytokine-containing CFC medium. Colonies were scored after 14 days (mean ± SEM, n = 4, Wilcoxon signed-rank test, *P = 0.0416, **P = 0.0078 in 133$^+$38$^-$, **P = 0.0011 in 133$^+$38^{int}, ***P = 0.004).

G, H Annexin staining for apoptotic cells was performed 2 days after transduction of human CB-CD34$^+$ exposed to an MOI of 100 PGK-GFP SIN LV (LV), MOI of 100 clinical-grade LV (clinical LV), MOI 10,000 of AAV6 and MOI 100 of PGK-GFP SINRV (RV) or to p24 equivalent of Env-less (Bald) or inactivated clinical LV as controls (Inactivated clinical LV) (mean ± SEM, n = 3, G), (mean ± SEM, n = 11 for Bald, LV and AAV6, n = 7 for RV, Dunn's adjusted Kruskal–Wallis test, *P ≤ 0.05; **P ≤ 0.01, H).

© 2017 The Authors

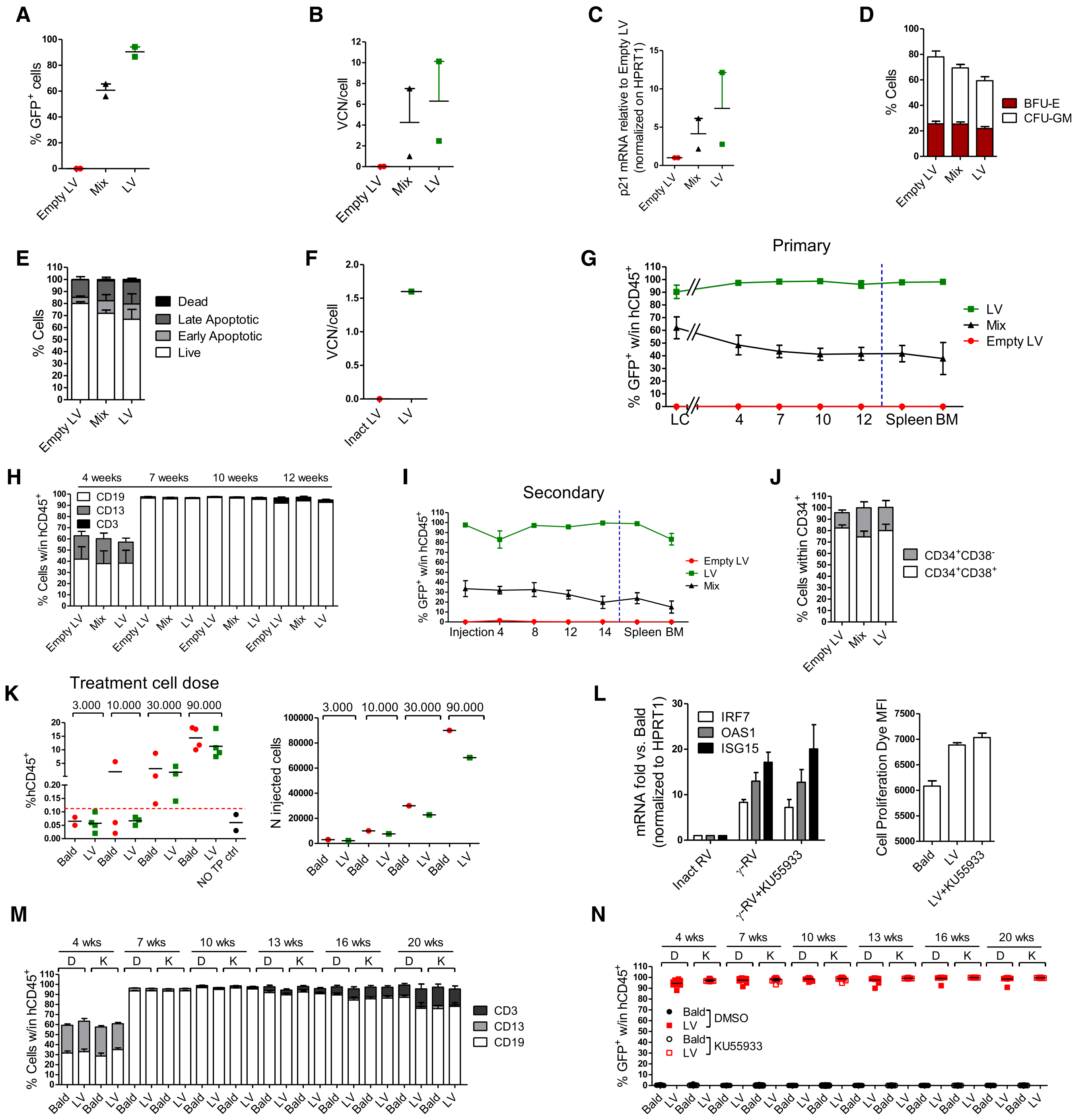

Figure EV3.

 © 2017 The Authors

Figure EV3. Liquid culture, CFU, and *in vivo* transduction of *in vivo* experiments.

A–E (A) Transduction efficiency at 5 days, (B) VCN at 14 days after (C) p21 mRNA induction (D) CFU counts and (E) Annexin V staining at 48 h after transduction of the cells that were transplanted in NSG Mice (Fig 3) (mean ± SEM, n = 2, A–E).

F VCN retrieved from the liquid culture of mPB-CD34$^+$ 14 days after transduction with the two-hit clinical standard protocol as described in Fig 3C (n = 1).

G–I Percentage of the GFP$^+$, and (H) myeloid (CD13$^+$), lymphoid (CD19$^+$ B, CD3$^+$ T) cells within the human CD45$^+$ cells in the peripheral blood over time, spleen and BM at the end of the experiment of the primary or secondary mice (mean ± SEM, n = 9 for LV and Mix, n = 10 for Empty LV, G, H) (mean ± SEM, n = 9 for LV, n = 10 for Empty LV and n = 11 for Mix, I).

J Frequencies of CD34$^+$38$^+$ and CD34$^+$CD38$^-$ within the human CD34$^+$ cells retrieved from the bone marrow of secondary recipients at the end of the experiment (mean ± SEM, n = 9 for LV, n = 10 for Empty LV and n = 11 for Mix).

K Percentages of human CD45$^+$ cells (left panel) detected in the bone marrow of NSG mice at 16 weeks after the transplant in the limiting dilution assay (LDA) experiment, and (right panel) the real number of CB-CD34$^+$ cells injected in the mice for the same experiment (each dot representing one mouse, n = 2 in Bald and n = 4 in LV for 3,000 cells, n = 3 for 10,000 and 30,000 cells, n = 4 for 90,000 cells, n = 2 for not transplanted controls, left panel) (each dot representing the number of cells injected in one mouse).

L Interferon-stimulated genes (left panel) mRNA levels in CB-CD34$^+$ 48 h after the transduction with an MOI 100 of PGK-SIN RV vector in the presence of KU55933 ATM inhibitor, (right panel) cell proliferation assay in CB CD34$^+$ 2 days after the transduction with MOI 100 of PGK LV in DMSO or KU55933 or MOI 100 of PGK RV or empty RV as control (mean ± SEM, n = 2).

M, N (M) Myeloid (CD13$^+$), lymphoid (CD19$^+$ B, CD3$^+$ T), and (N) GFP$^+$ cells within the human CD45$^+$ cells were monitored in the peripheral blood over time (mean ± SEM, n = 8 for Bald DMSO, n = 10 for LV DMSO, n = 11 for Bald and LV KU55933, M) (each dot representing one mouse, n = 8 for Bald DMSO, n = 10 for LV DMSO, n = 11 for Bald and LV KU55933, N). D = DMSO, K = KU55933.

© 2017 The Authors
