# Supplementary figures and images for: Lentiviral vectors escape innate sensing but trigger p53 in human hematopoietic stem and progenitor cells
Source: EMBO Mol Med. 2017 Jun 30;9(9):1198–211. doi: 10.15252/emmm.201707922 (PMC5582409; doi:10.15252/emmm.201707922)

Source Data, WB, Fig EV1J

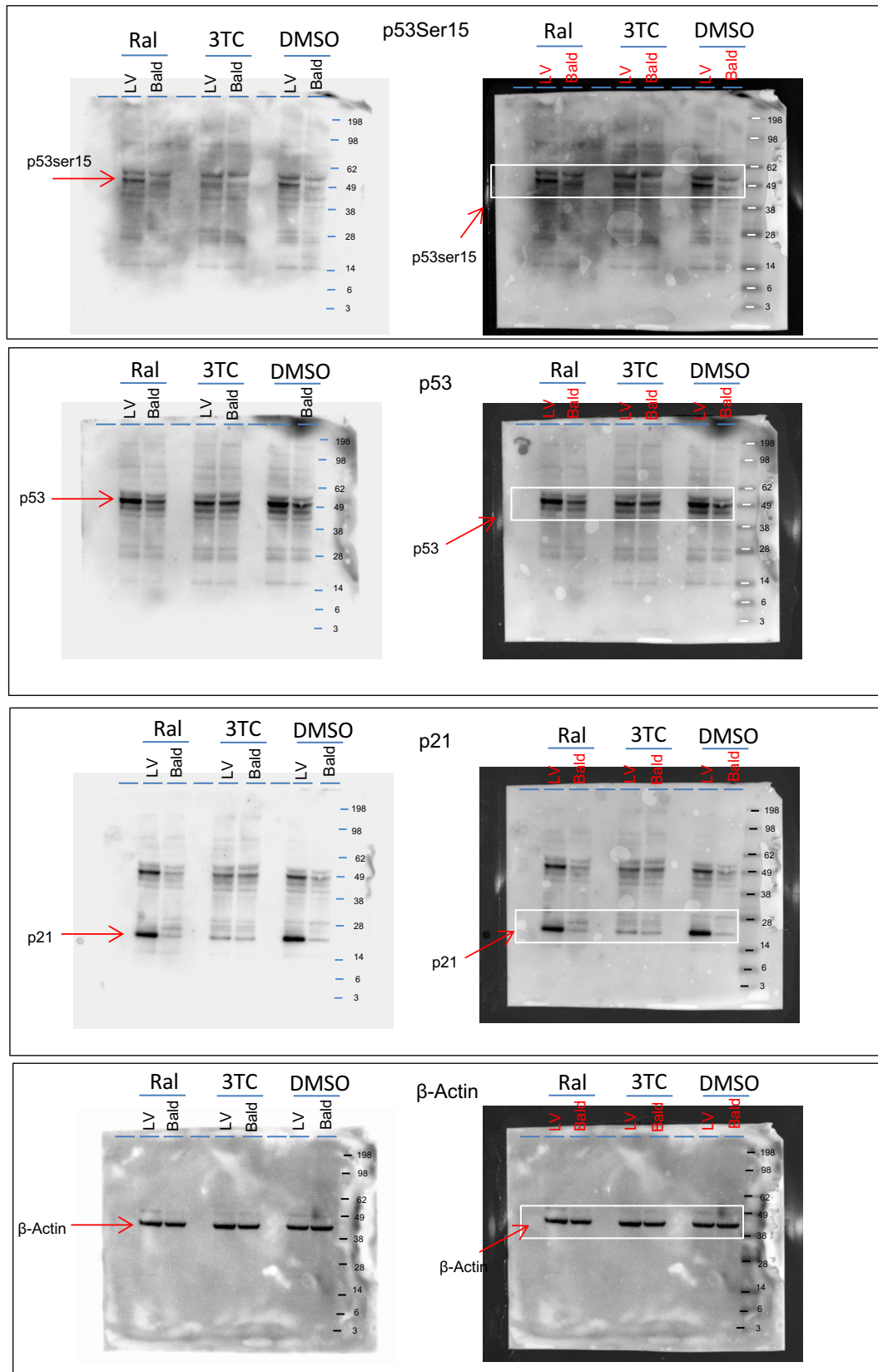

Supplement: Supplementary file 3 — Source Data for Expanded View [file EMMM-9-1198-s005.zip › Source_data_for_Figures_EV1J/Source_data_for_Figures_EV1J.pdf]

Source Data, WB, Fig 1J and Fig 4D

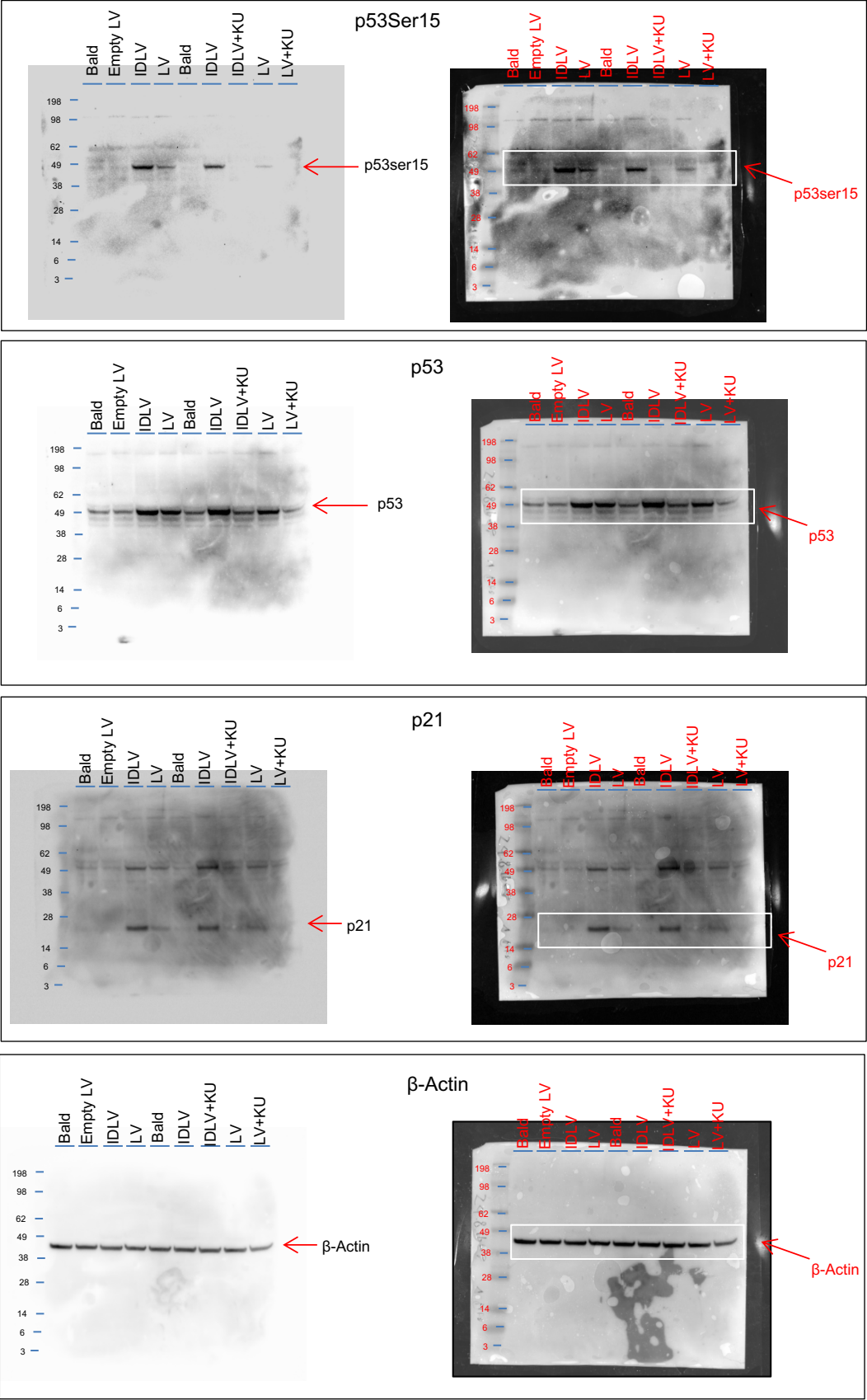

Supplement: Supplementary file 5 — Source Data for Figure 1 [file EMMM-9-1198-s003.pdf]
